# *H. pylori*-infection and antibody immune response in a rural Tanzanian population

**DOI:** 10.1186/1750-9378-1-3

**Published:** 2006-09-14

**Authors:** Sam M Mbulaiteye, Benjamin D Gold, Ruth M Pfeiffer, Glen R Brubaker, John Shao, Robert J Biggar, Michie Hisada

**Affiliations:** 1 Division of Cancer Epidemiology and Genetics, National Cancer Institute, NIH, Department of Health and Human Services, Rockville, Maryland, USA; 2 Division of Pediatric Gastroenterology and Nutrition, Department of Pediatrics, Emory University School of Medicine, Atlanta, Georgia, USA; 3 Medical Advisor, Interchurch Medical Assistance; New Windsor, Maryland, USA; Formerly, Director, Shirati Hospital, North Mara District, Tanzania; 4 Department of Medical Microbiology and Immunology, Kilimanjaro Christian Medical Center, Moshi, Tanzania

## Abstract

**Background:**

*Helicobacter pylori *(*H. pylori*) infection is ubiquitous in sub-Saharan Africa, but paradoxically gastric cancer is rare.

**Methods:**

Sera collected during a household-based survey in rural Tanzania in 1985 were tested for anti-*H. pylori *IgG and IgG subclass antibodies by enzyme immunoassay. Odds ratios (OR) and confidence intervals (CI) of association of seropositivity with demographic variables were computed by logistic regression models.

**Results:**

Of 788 participants, 513 were aged ≤17 years. *H. pylori *seropositivity increased from 76% at 0–4 years to 99% by ≥18 years of age. Seropositivity was associated with age (OR 11.5, 95% CI 4.2–31.4 for 10–17 vs. 0–4 years), higher birth-order (11.1; 3.6–34.1 for ≥3^rd ^vs. 1^st ^born), and having a seropositive next-older sibling (2.7; 0.9–8.3). Median values of IgG subclass were 7.2 for IgG1 and 2.0 for IgG2. The median IgG1/IgG2 ratio was 3.1 (IQR: 1.7–5.6), consistent with a Th2-dominant immune profile. Th2-dominant response was more frequent in children than adults (OR 2.4, 95% CI 1.3–4.4).

**Conclusion:**

*H. pylori *seropositivity was highly prevalent in Tanzania and the immunological response was Th2-dominant. Th2-dominant immune response, possibly caused by concurrent bacterial or parasitic infections, could explain, in part, the lower risk of *H. pylori*-associated gastric cancer in Africa.

## Background

In sub-Saharan Africa, *Helicobacter pylori *(*H. pylori*) infection is ubiquitous, with seroprevalence reaching 90% or higher in many populations [1]. *H. pylori *is transmitted from person-to-person, and transmission risk is high in populations of low socioeconomic status, poor hygiene, and limited access to clean water [2-4]. The most severe consequence of chronic *H. pylori *infection is gastric adenocarcinoma [1]. However, gastric cancer rates vary widely worldwide and correlate imperfectly with *H. pylori *seroprevalence [5]. For example, seroprevalence reaches 80% by 5 years of age in sub-Saharan Africa [6,7], highlighting the particularly young age of infection acquisition, and therefore duration of infection. However, age-standardized gastric cancer incidence rates are relatively low at 2–21 per 100,000 person-years for both males and females [8]. 

In Japan, seroprevalence increases more gradually with age to a prevalence of 40% to 70% among adults [9,10], but the age-standardized gastric cancer incidence rates are substantially higher, ranging from 65–92 and 24–39 per 100,000 person-years among males and females, respectively [8]. By comparison, seroprevalence estimates in the U.S. range from 10 to 20% among adults [10,11], with the age-standardized gastric cancer incidence rates being 6.6 and 2.6 per 100,000 person-years among white males and females, respectively [8]. 

These statistics highlight a paradoxical deficit of gastric cancer cases in sub-Saharan Africa, compared to Western countries after controlling for age, the so-called "African enigma" [12]. Gastric cancer deficits may be artifactual, due to incomplete case ascertainment and competing mortality [13,14]; however, those reasons do not explain why gastric cancer rates vary in African populations that have comparable access to medical care. Specifically, populations residing in mountainous areas tend to have a higher relative frequency of gastric cancer as compared to populations residing in lowland areas [15].

Variation in gastric cancer rates within Africa, and elsewhere, suggests the presence of modifying factors on *H. pylori*-associated gastric cancer risk. In other words, outcomes of *H. pylori *infection could be influenced by bacterial, host, diet, or other environmental factors. One hypothesis, based on animal studies, posits that bacterial and/or parasitic infections modulate *H. pylori*-induced gastric cancer risk [16], perhaps by altering the quality of *H. pylori*-induced mucosal immunity in the stomach [17,18]. *H. pylori *infection is thought to cause gastric cancer by eliciting vigorous T-helper (Th1) pro-inflammatory cellular immune responses in gastric mucosa [16] and the resulting mucosal injury is mediated by pro-inflammatory cytokines and oxygen radicals secreted by infiltrating chronic inflammatory cells [16,19]. Parasites and, to a lesser extent, certain bacterial infections [18], elicit Th2 instead of Th1-dominant immune responses to thwart their elimination [20] and could plausibly modulate *H. pylori*-induced immune response towards one less damaging to the gastric mucosa.

We hypothesized that persons living in high *H. pylori*-prevalence areas with low gastric-cancer incidence in Africa would therefore have Th2-type dominant *H. pylori*-specific responses. To test this hypothesis, we evaluated *H. pylori *seropositivity and *H. pylori*-specific IgG subclass antibodies in a rural population in northern Tanzania, where *H. pylori *infection was expected to be endemic and gastric cancer incidence is thought to be low.

## Subject selection and serological methods

The study subjects were residents of the North Mara District, located on the eastern shores of Lake Victoria in Tanzania, who participated in human immunodeficiency virus (HIV) serological surveys from May through June, 1985 [21]. Participants provided individual verbal consent, and parents provided verbal consent for their children, to participate in serological surveys. Institutional Review Boards gave ethical approval for the study. Participants were recruited from households, defined as compounds where individuals shared meals and had one person designated as head. As previously observed [21], polygamy was frequently practiced in this population and headmen often had several wives. The households were randomly selected from nine villages located either on hills (n = 5) or in valleys (n = 4). Participants provided sociodemographic information and gave a blood sample [21]. The samples were stored at -80°C and were thawed once before current testing. Samples from all but 10 participants enrolled in the original study were available for *H. pylori *serologic testing.

Anti-*H. pylori *antibodies were measured using an IgG enzyme immunoassay (EIA) as previously reported [22-24]. This assay has been validated in various populations, including those from Africa and was shown to have high sensitivity (89–96%) and specificity (92–97%), using biopsy-proven *H. pylori *gastric mucosa infection as the gold standard [22,23]. The tests were run in triplicate using a standard 96-well microtiter plate and placing the plates on a Benchmark microplate reader (BioRad, Hercules, CA). EIA cut-off values were derived using known *H. pylori*-positive and negative control sera in which OD values < 0.8 were considered to be negative, OD values >1.3 were considered positive, and OD values between 0.8 and 1.3 were considered to be indeterminate, as previously described [22,24]. To determine the IgG antibody subclasses (IgG1 or IgG2) in seropositive individuals, as a marker of Th1/2-type cellular response [25], we used IgG subclass EIAs to mouse anti-human IgG1 and mouse anti-human IgG2 conjugated to HRP (Zymed Laboratory, San Francisco, CA). In brief, pooled serum was used as a reference standard for the IgG subclasses. This standard was titrated using each of the IgG subclass antibodies (i.e. mouse anti human IgG1 and mouse anti human IgG2) to determine the highest dilution at which reactivity could still be detected and also remain linear on a standard curve [22,23,26]. The maximal dilution for both IgG1 and IgG2 subclasses was 1:25,600. The serum was assigned an arbitrary ELISA unit of 1 at this dilution. The ELISA was performed as previously stated using positive controls diluted to 1:25,600 based on the previously mentioned standard curve and double the concentration at 1:12,800 dilution. The IgG subclass unit was calculated as ratio of the OD_490 _of the individual sample to the OD_490 _of the standard (i.e., IgG subclass unit = sample OD_490_/reference OD_490_). These assays were modified to determine the optimal concentration of antigen in serum and IgG conjugate that discriminated between *H. pylori*-positive and *H. pylori*-negative samples. Each plate incorporated *H. pylori*-specific IgG subclass positive and negative control samples. OD values for IgG1 and IgG2 subclasses were normalized using standards previously reported [22]. We calculated the ratio of IgG1/IgG2 subclasses to determine phenotype of Th1/2-type cellular immune response. Using a previously established criteria to determine Th1 vs. Th2-type response in African and western populations [27,28], an IgG1/IgG2 ratio >1.0 suggests a Th2-(IgG1)-dominant cellular immune response and a ratio ≤ 1.0 suggests a Th1-(IgG2)-dominant response.

### Statistical analysis

Associations between sociodemographic variables (sex, age, village location) and *H. pylori *seropositivity were determined using Chi-square or Fisher's exact tests. Because of the high *H. pylori *seroprevalence, we grouped persons with indeterminate status with seronegative individuals for the analysis. Odds ratios (OR) and 95% confidence intervals (CI) of association of *H. pylori *seropositivity with age, sex, birth-order were estimated using logistic regression models (PROC GENMOD in SAS 9.1 software package; SAS Institute). Because children are more likely to acquire infection from an infected older sibling, we also estimated the association between seropositivity of a younger child with status of the next-older sibling on whom samples were available. We accounted for intra-familial or intra-household correlations among observations by using generalized estimating equations (25). We used independence working correlation matrices in the computations and checked results by also using equi-correlated working correlations that assumed that members in the same family would have equal correlations. Both methods yielded similar results, so only the results using independent working correlations are presented. Values of IgG1/IgG2 ratio among seropositive individuals were log-transformed to obtain a normal distribution. We assessed the relationship between the log-transformed IgG1/IgG2 ratio and age in linear regression models. Age was used in the categories 0–4, 5–9, 10–17, and 18+ years. A two-sided p-value < 0.05 was considered statistically significant.

## Results

Of 788 participants, 351 (44%) were male. The majority 513 (65%) were 17 years old or younger (Table 1). Only 2 subjects were HIV-positive, so this variable was not analyzed further. Overall, 725 (92%) subjects were seropositive for *H. pylori *antibody; 27 (3%) were seronegative and 36 (5%) were indeterminate. *H. pylori *seropositivity rose steeply with age from 76% in children aged 0–4 years to 99% in adults (p_trend _< 0.001; Table 1). *H. pylori *seropositivity was similar among males and females. Among children, those aged 10–17 years were more likely to be *H. pylori *seropositive compared to those aged 0–4 years (OR 11.5, 95% CI 4.2–31.4; Table 1). Similarly, children of higher birth-order (≥3) were more likely to be seropositive compared to first-born children (OR 11.1, 95% CI 3.6–34.1; Table 1). In a multivariable model, both age group and birth-order were independently associated with *H. pylori *seropositivity (OR 6.3, 95% CI 1.3–31, for 10–17 vs. 0–4 years and OR 4.0, 95% CI 1.1–15, for ≥3^rd ^vs. 1^st ^born). *H. pylori *seropositivity was marginally higher in children with a seropositive next-older sibling compared to those whose next-older sibling was seronegative (OR 2.7, 95% CI 0.9–8.3). All 62 married men and their 113 wives were *H. pylori *seropositive. Their concordant results precluded us from evaluating the association of seropositivity between spouses and within parent-child units. *H. pylori *seropositivity was not associated with location of the village in valleys (OR 0.66; 95% CI 0.33–1.33).

**Table 1:** Frequency and risk of *H. pylori *seropositivity among persons residing in rural Tanzania (May to June, 1985)

Characteristic	No. *H. pylori *positive/Total No. (%)	OR	95% CI	P value
Sex *				0.87
Female	402/436 (92%)	Ref.		
Male	322/351 (92%)	1.04	0.67–1.62	
				
Age group, years*				<0.001
0–4	138/181 (76%)	Ref.		
5–9	168/180 (93%)	4.4	2.42–7.81	
10–17	148/152 (97%)	11.5	4.22–31.4	
≥18	264/268 (99%)	20.6	7.57–56.2	
				
Birth order†				<0.001
1	79/105 (75%)	Ref.		
2	82/101 (91%)	3.3	1.7–6.8	
≥3	136/139 (97%)	11.1	3.7–34	
				
Next-older sibling ±				0.08
Seronegative	23/29 (79%)	Ref.		
Seropositive	189/208 (91)	2.70	0.90–8.34	
				
Location of village				0.25
Hill	272/290 (94%)	Ref.		
Valley	453/498 (91%)	0.66	0.33–1.33	

Abbreviations: OR odds ratio; CI Confidence Interval

* Sex is missing for one person and age is missing for 7 persons

† Only children with information on birth-order included in this analysis

± "Next-older sibling" refers to the next-older sibling of the case child

**Figure 1:**
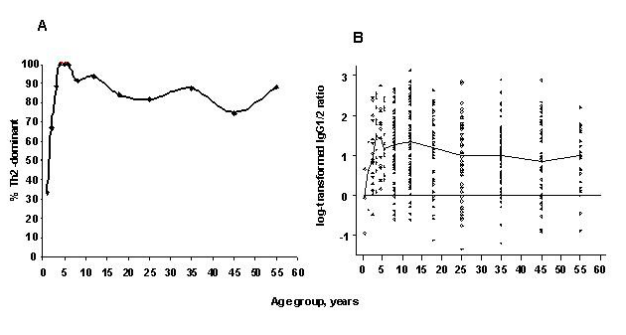
Th2 type response by age group, in years: **Panel A**: Percent of *H. pylori *seropositive individuals with Th2-dominant IgG *H. pylori *specific immune response by age group. **Panel B**: Log-transformed IgG1/IgG2 ratio by age group among *H. pylori *seropositive individuals in rural Tanzania. The X-axis shows age groups in years, the Y-axis shows log-transformed values. The y-line marks the cut-off value for T helper (Th)-dominant 1 vs. Th2-dominant response: values above the line indicate Th2-dominant responses, while values below the line indicate Th1-dominant responses. The line plot connects the median log-transformed IgG1/2 ratios for each age group and the scattered points indicate actual values for each age group.

Among seropositive individuals, the median values for the IgG subclass antibodies were IgG1: 7.2 (inter-quartile range [IQR]: 3.6–12.4) and IgG2: 2.0 (IQR: 1.4–3.2). The median IgG1/IgG2 ratio was 3.1 (IQR: 1.7–5.6). Children were more likely to have an IgG1/IgG2 ratio, consistent with Th2-dominant immune response, compared with adults (OR 2.4, 95% CI 1.3–4.4). In analyses excluding seronegative individuals, the proportion of persons showing Th2-dominant responses increased from 33% among children aged 1 year to 100% among children 3–6 years and then declined somewhat to 80% among subjects aged 45 years and older (Figure 1, panel A). Similarly, values of the log-transformed IgG1/IgG2 ratio rapidly increased with age to peak between 3–4 years and then reached a plateau in the adult years (Figure 1, panel B). Models of the log-transformed IgG1/IgG2 ratio that included age as a quadratic did not fit the data better than models with linear age (Pearson χ^2^-test = 0.72). The IgG1/IgG2 ratio was unrelated to gender, birth order, or village location (data not shown).

## Discussion

We observed extremely high *H. pylori *seroprevalence in a rural population in northern Tanzania. Seropositive individuals showed a high IgG1/IgG2 ratio, suggestive of Th2-dominant *H. pylori*-specific immune responses. Our cross-sectional study confirms high *H. pylori *seropositivity in rural Tanzania, and shows that seropositivity increases steeply with age in childhood and is associated, among children, with higher birth-order and with having a seropositive next-older sibling. These findings are consistent with prior studies of *H. pylori *infection in highly endemic areas [2,5]. A novel finding from our study is the Th2-dominant *H. pylori*-specific immune responses, which were strongest in children but were present also in adults. As assessed by IgG subclass antibodies, the Th2-dominant immune responses to *H. pylori *infection observed in this rural Tanzanian population contrasts to Th1-dominant responses reported in Western populations [27,29]. We speculate that Th2-dominant *H. pylori*-specific immune responses in this population can be modulated by concurrent infection with parasitic or bacterial infections as has been observed in animal models [16,18]. Th2-dominance rapidly increased with age and peaked between 3–7 years. This trend with age may reflect a tendency for younger children to have a Th2-dominant pattern or may be due to a high frequency and burden of parasitic and bacterial infections among children, or both. Parasite egg and/or worm burden for schistosomiasis and soil-transmitted helminths have been shown to peak at young ages in this population [30].

Our study highlights one aspect of the gastric cancer paradox in Africa, i.e., early acquisition of *H. pylori *infection in childhood and persistence of seropositivity into adulthood in an area where gastric cancer is relatively rare. Competing mortality (causing deficits in the elderly population ≥ 60 years) has been advanced as one explanation for the lower age-sex specific gastric cancer incidence in sub-Saharan Africa compared to developed countries. However, this explanation is not supported by data from South Africa where life expectancy prior to the AIDS epidemic was 63 years [31]. Investigators at Shirati Hospital, North Mara District, who serve our study population and are actively engaged in cancer research [32], previously reported gastric cancer to be rare. In an analysis of 279 malignancies seen at the Hospital from 1952–1965, only 12 (4.3%) were diagnosed as gastric cancer [33]. Conversely, gastric cancer is relatively more frequent on the slopes of Mount Kilimanjaro, where it contributes ~15% of malignancies [34]. Moreover, there are similar reports of higher gastric cancer rates in mountainous or dry areas elsewhere in Africa [15,35,36]. The variation in relative frequency of gastric cancer in the context of similar *H. pylori *prevalence, similar access to medical care, and similar life expectancy has led to the hypothesis that environmental co-factors, perhaps diet (nitrates or fresh fruits and vegetables), are important. However, no dietary factors have been convincingly implicated in Africa. An alternative hypothesis posits that enteric or other parasites may influence gastric cancer risk [16]. This hypothesis is based on the observations in mice that *Helicobacter*-induced gastric atrophy, a precursor lesion to gastric cancer, improved in mice concurrently infected with enteric parasites but not in mice that were not. Improvements were associated with concomitant down-regulation of the pro-inflammatory responses due to a shift from Th1 toward Th2-dominant cellular immune response in mice with concurrent enteric parasite infection [16]. Our finding of Th2-dominant immune response in *H. pylori *infected persons from northern rural Tanzania, where gastric cancer is relatively rare, is consistent with this hypothesis. Although we lack data on parasite or colonic bacterial infections, it is reasonable to assume that people who live in remote rural African villages without access to running water or other amenities, such as ours, are frequently infected with parasitic and/or bacterial conditions in addition to *H. pylori *[30,37].

The role of Th1/2 immunity and *H. pylori*-induced gastric cancer has been investigated in two prior studies. In one conducted in Soweto, South Africa, where gastric cancer incidence is low, found Th2-dominant *H. pylori*-specific immune responses in blacks with gastric symptoms [27] whereas study of symptomatic white subjects from Austria and Germany, where gastric cancer incidence is high, demonstrated Th1-dominant responses [27]. The authors suggested that the Th2-dominant responses in blacks were likely induced by co-infection with parasites, which they postulated may modulate gastric risk cancer among Africans. In the other study conducted in Columbia in persons from low and mountainous regions where gastric cancer incidence is low and high, respectively, evaluated *H. pylori *seropositivity and parasitic infections. Persons from Tumaco, a low lying area where gastric cancer incidence is low, had Th2-dominant immune responses and a higher prevalence of helminth infections [28]. Conversely, those from Pasto, a high altitude area where gastric cancer incidence is high, had predominantly Th1-dominant immune responses and a lower prevalence of helminth infections [28]. The authors attributed differences in Th1 or Th2-dominant responses across the low vs. mountainous regions to differences the prevalence of helminth infections and suggested that parasitic infection may modify *H. Pylori*-induced gastric cancer risk in Columbia. Thus, those studies and ours lend support to the hypothesis that Th1/2 immunity modulated by parasite infections may influence the risk of *H. pylori*-associated gastric cancer in diverse environments [15,28,35,36].

Our study has some limitations. *H. pylori *infection was measured using serological assays, and some subjects may have been misclassified. The finding of almost universal seropositivity among adults is surprising because we would have expected a small proportion of individuals to be seronegative due to loss of bacterial colonization that occurs with age in chronically infected persons or with development of chronic gastric atrophy. We lacked endoscopy data on the state of gastric mucosa (presence of ulcer, gastric atrophy or not) and therefore are unable to draw conclusions about the relationship between Th2 dominance and severity of gastric mucosal inflammation. Direct measurement of pepsinogen levels could have also provided some information on the presence or not of gastric atrophy, but the volume of residual samples from the study population were inadequate for additional testing. Furthermore, our study was cross-sectional so we cannot infer temporality of associations demonstrated. We note that our "highland" area villages were in low hills, not mountainous areas, thus our study does not provide data for Th1 vs. Th2 responses in low vs. mountainous regions in Africa. The use of IgG subclasses assays as markers for type1/2-dominant immune response in a general population in Africa is novel, and our study provides valuable baseline data for sub-Saharan Africa. Finally, IgE measurements would strengthen our results on the role of parasites, but we lacked a validated assay to perform IgE studies.

To conclude, *H. pylori *seropositivity was highly prevalent in rural Tanzania. Seropositive persons showed a Th2-dominant immune response to *H. pylori *infection, which may be due to effects of concurrent infection with parasites and/or bacterial infections. We speculate that the shifts in immune response from Th1 responses to Th2 responses in early childhood and persistence of that immune profile into adulthood may partially explain the paradoxically lower gastric cancer risk in highly *H. pylori*-endemic Africa.

## Competing interests

The author(s) declare that they have no competing interests.

## Authors' contributions

SMM conceived of the study, contributed to analysis and interpretation of data, and drafted the manuscript. BDG carried out the serological studies, contributed to interpretation of data and writing the report. RMP carried out the statistical analyses and interpreted data. GRB and JCS did the field work, contributed to interpretation of data. RJB participated in the design of the study, field work, and contributed to analysis and edited the manuscript. MH participated in study design and coordination and helped with analysis, interpretation of data, and editing the manuscript. All authors read and approved the final manuscript.
